# Tooth discoloration and internal bleaching after the use of ledermix paste with various bleaching agents – An *in vitro* study

**DOI:** 10.4317/jced.55195

**Published:** 2018-11-01

**Authors:** Asokan Yogha-Padhma, Athikesavan Jayasenthil, Ramaraj Pandeeswaran

**Affiliations:** 1 Postgraduate student, Department of conservative dentistry and endodontics, Adhiparasakthi dental college and hospital, Melmaruvathur; 2 MDS, Reader, Department of conservative dentistry and endodontics, Asan Memorial dental college and hospital, Chengalpattu; 3 BDS, Postgraduate student, Department of orthodontics, JKK Nataraja dental college and hospital, Kumarapalayam

## Abstract

**Background:**

To assess the reversal of discolouration caused by Ledermix using various bleaching agents.

**Material and Methods:**

Twenty six extracted human mandibular premolars were taken and divided into four groups. Six teeth were divided into three each which are taken as positive and negative controls. The remaining twenty are divided into two groups (n=10). After conventional access preparation, the Ledermix paste was sealed in the pulp chamber for twelve weeks. The paste was removed by a rinse with sodium hypochlorite (NaOCl). Then the pulp chamber was sealed with a mixture of Sodium perborate and distilled water for group 1 and group 2 was sealed with Sodium tetraborate for 1 to 12 weeks. The shade was measured by a Spectrophotometer at four time periods baseline(T0), after 12 weeks of placement of Ledermix (T1), after 4 (T2), 12 (T3) weeks of Internal bleaching with Sodium perborate and Sodium tetraborate respectively. Data were collected based on CIE-76 (L*a*b*) system and analysed using t-test and ANOVA.

**Results:**

A significant decrease in the mean value of L*(lightness) was observed after treatment with Ledermix (T1, *p*<0.05). Considerable increase in these values after bleaching with Sodium perborate and Sodium tetraborate (T2, T3) were found in both groups, to the same extent.

**Conclusions:**

Ledermix discoloured the tooth structure but discolouration could be reversed when bleached with both Sodium perborate and Sodium tetraborate to the same extent.

** Key words:**Bleaching, discoloration, ledermix, sodium perborate, sodium tetraborate.

## Introduction

The ultimate goals of endodontic treatment are to remove as many bacteria, their by-products, and pulpal remnants from the infected root canal system and then to create an preparation with antimicrobial agents, such as chemical irrigants or intracanal dressings, environment in which any remaining organisms cannot survive by sealing the disinfected root canals completely (1).

Microbial invasion is time related and bacterial species dependent. Hence, early endodontic treatment of a tooth should minimize the number of micro-organisms lodged in the dentinal tubules. Micro-organisms in dentinal tubules may constitute a reservoir from which root canal and surrounding tissue infection and re-infection may occur (2). Therefore, endodontic medicaments must be able to penetrate into dentinal tubules and kill bacteria within them (3).

Medicaments that combine antibiotics and corticosteroids elements are highly effective in root canal therapy. The corticosteroid constituent reduces periapical inflammation and gives almost instant relief of pain and reduces inflammation of the periapical region of the patient who complaints of extreme tenderness to percussion after the canal instrumentation and the antimicrobial properties are catered by antibiotics (4).

Ledermix paste is an endodontic medicament used in many parts of the world. The main therapeutic components are triamcinolone acetonide (1% - a corticosteroid) and demethylchlortetracycline (3.021% - also known as demeclocycline, a tetracycline antibiotic) (5). Although there have been controversies surrounding the use of Ledermix paste, studies have shown the placement of Ledermix reduced the incidence of pain following initial canal debridement and also studies have shown that Ledermix causes discoloration of teeth (6,7).

A discolouration is any change in colour of a tooth, either externally or internally, that presents a major aesthetic problem, especially if it involves the anterior teeth (8). The aesthetic appearance of a treated tooth still concerns the clinician and significantly affects the patient’s quality of life (9). To overcome the discolouration internal bleaching procedures can be carried out. Internal bleaching procedures such as the walking bleach technique can be used for whitening of discoloured root filled teeth. The walking bleach technique is performed by application of a paste consisting of Sodium perborate- (tetraborate) and distilled water (3% H2O2), respectively, in the pulp chamber (10).

The aim of this study was to assess the reversal of discolouration caused by Ledermix using various bleaching agents.

## Material and Methods

-Sample selection

Twenty six extracted human mandibular premolars with closed apices were used. All teeth were cleansed and then radiographed to verify the absence of resorption, dental caries, previous endodontic treatment, cracks or root fractures. The teeth were sterilized in an autoclave and were immersed in saline for hydration in individually labelled bottles for 72 hours.

-Sample preparation

The access opening was achieved, and all contents of the pulp chamber and coronal portion of the root canal were removed with a size 15 hand file and distilled water. A cotton pellet was placed in the pulp chamber and the access preparation was sealed with Cavit (3M, Espe).

-Shade recording

Shade recording of the crown was performed with a Minolta spectrophotometer CM-3600d. The equipment quantifies the shade through a system named CIE-76 (L*a*b*). The L* value indicates lightness and varies between 0 for black and 100 for white; a* determines the amount of red (positive values) or green (negative values); and b* exhibit the amount of yellow (positive values) or blue (negative values).

The shade was measured in the same room under the same light by one calibrated operator at four different time periods; baseline (T1), after 12 weeks of placement of Ledermix (T2), after 4 weeks of placement of Sodium perborate / Sodium tetraborate in the pulp chamber (T2), 12 weeks after placement of Sodium perborate / Sodium tetraborate (T3).

At each time period, three measurements of L*, a*, b* were recorded and ΔL, Δa, Δb were calculated by subtracting the final data from the initial data within each time period. The constant difference in colour (ΔE) was then calculated using the formula: ΔE= [(ΔL)2+ (Δa)2+ (Δb)2].

-Experimental procedures

The premolars were divided into four categories. Six teeth were divided into three each which are made as positive and negative controls. The remaining twenty specimens were divided and assigned into two experimental groups (n=10).

A baseline shade was taken before any material was placed in the pulp chamber(T0). The Cavit [3M,Espe] and the cotton pellet were removed, and the Ledermix paste was mixed with distilled water to a thick consistency. The mixture was placed in the pulp chamber with a plastic instrument and the access cavity was again sealed with Cavit (3M,Espe). Twelve weeks later, the tooth shade was recorded (T1). The Cavit (3M,Espe) was removed, and the medicament was rinsed out of the tooth with 5ml of 2.5% NaOCl. The pulp chamber was inspected to ensure that the medicament had been completely removed and the shade was again recorded (T2).

For the bleaching procedure, Sodium perborate powder with distilled water and Sodium tetraborate with distilled water (2g:1ml ratio) to a thick consistency, placed in the pulp chamber with a plastic instrument and then sealed with Cavit (3M, Espe) for 4 weeks, for group 1 and group 2 respectively. After 4 weeks, the temporary filling is removed and the pulp chamber was thoroughly rinsed with 5ml of 2.5% NaOCl and fresh Sodium perborate / Sodium tetraborate pastes are placed in pulp chambers of group 1 and group 2 and sealed for 8 additional weeks after which the final shade (T4) was taken.

-Statistical analysis

The data were analysed at 5% significance. The t-test was used to analyse the intergroup data: the colour stability [ΔE] of the two experimental groups was compared at each time period. ANOVA test were used for intragroup comparisons.

## Results

Discolouration was observed visually in all specimens after the Ledermix was sealed in the pulp chamber for 12 weeks. Improvement of the discolouration was noted in time periods T2 and T3, after removal of the Ledermix and when bleaching agent was carried out.

In the intragroup analysis, the values of lightness (L*) were considered at different time periods. There was a significant drop in the values of L* (between T0 and T1, in both groups). The values rose over the subsequent time periods, increasing significantly in both groups by T3 and reaching a shade close to the one at T0 at T3 ( Table 1).

**Table 1:**
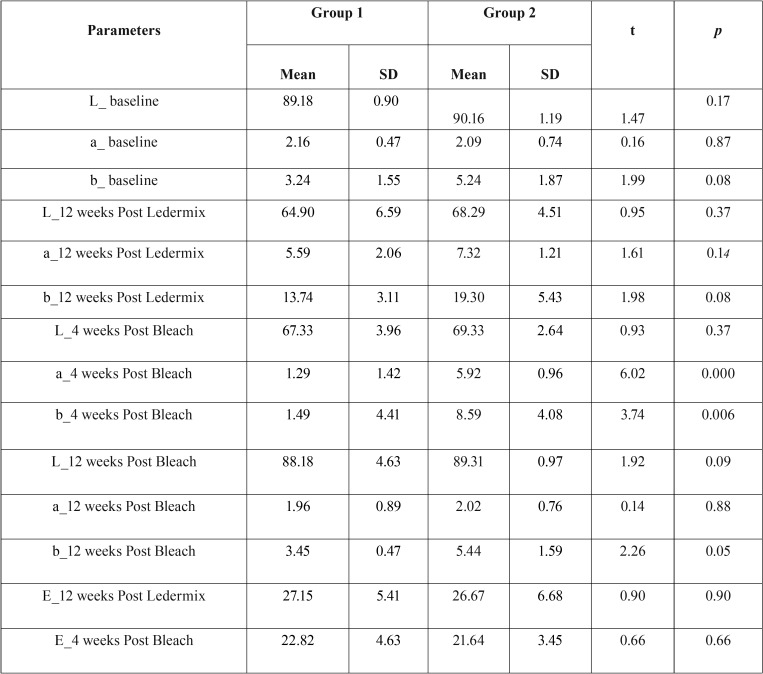
Mean values of L*,a*,b*and ΔE (±SD) at T0, T1, T2 and T3.

In the intergroup analysis, the overall colour stability was considered (ΔE). The patterns of the discolouration and reversal of discolouration due to bleaching with two different agents were similar between group 1 and 2.

## Discussion

Micro-organisms are the main etiological agents of pulp and periradicular diseases. However with proceeding infection, necrosis and apical periodontitis the entire root canal system becomes invaded by bacteria (11). The rationale behind intracanal antiseptic medication is to eliminate bacteria from the root canal and prevent reinfection (12).

An antibiotic/corticosteroid combination, Ledermix has been extensively used in various parts of the world in paste and cement forms as both a vital pulp dressing and a root canal medicament.This study confirmed that Ledermix discoloured the teeth, as the L* values decreased drastically between T0 and T1. Tetracycline is the antibiotic in the Ledermix paste responsible for tooth discolouration. Tetracycline has the ability to chelate calcium ions and to be incorporated into the teeth, resulting in discoluration. The exact mechanism of tetracycline staining however is still unknown. However, Ledermix is an effective anti-inflammatory action paste.

The standard procedure of rinsing off the Ledermix paste with NaOcl would have been sufficient to improve the discoloration but the oxidation reaction may not have been powerful enough to do so either. Seung Tae Kim *et al.* said about the effects of Ledermix paste as an intracanal medicament on the discolouration of teeth, but no study yetassessed whether bleaching procedures could reverse the discolouration caused by the Ledermix. Bleaching with Sodium perborate and Sodium tetraborate paste, mimicking the walking bleach technique for time periods (T2 and T3) improved the discolouration of the teeth. When in contact with moisture, Sodium perborate will slowly decompose into Sodium metaborate, hydrogen peroxide and singlet oxygen, which will bleach the dentine by a simple oxidation-reduction reaction. When sodium tetraborate is mixed with water it decomposes to hydrogen peroxide which helps in bleaching.

Clinically, the walking bleach technique is a simple procedure with results that have a high patient acceptance. (Gupta and Saxena 2014) (13). In this study, teeth were bleached with Sodium perborate and Sodium tetraborate to assess whether there would be difference in the patterns of reversal of discoloration by bleaching.

Teeth in both group 1 and group 2 at T2 exhibits a very mild increase in value of L* from T1, but the value of L* increases drastically and more closely to the values of L* at baseline at T3. Therefore, at T3 the L* is more or less similar to L* at T0, proving that the discolouration caused due to Ledermix paste could be reversed by internal bleaching procedures with Sodium perborate and Sodium tetraborate to the same extent.

In this study, spectrophotometry based on the CIE L*a*b* system was used to assess changes in shade. The advantage of this system is that the shade difference are expressed numerically, and these can be related to visual perception and clinical significance (14). Values of L*a* and b* were collected, as they were needed in the formula used to calculate the ΔE, which represents the constant difference in colour, regardless of the location in the colour space (15).

Values at different time periods have to be collected to calculate aΔ. aΔ value is calculated by subtracting the final data from the initial data in each time period. It was possible to calculate the constant difference in colour (ΔE) for intergroup comparisons, where characteristics were compared between two time periods. However, only values of L* (lightness) were considered for intragroup comparison as Δ could not be calculated. As the darkening and lightening patterns were the ones of biggest concern, this does not represent a limitation (16).

This present study had shown that severe discoloration was caused by Ledermix when used as an intracanal medicament. Also, the discolouration caused by Ledermix can be reversed to a shade similar to the shade at baseline, indicating both the bleaching agents’ Sodium perborate and Sodium tetraborate have similar bleaching efficiency to bring back the discoloured teeth as like before.

## Conclusions

Within the limitations of the study, Ledermix discoloured the tooth structure and the same tooth could be bleached with Sodium perborate and Sodium tetraborate paste. Despite the possible starring effects, Ledermix paste should not be excluded by the clinicians when choosing endodontic medicaments as there are many advantages associated with the material.
